# Transcriptional role of androgen receptor in the expression of long non-coding RNA Sox2OT in neurogenesis

**DOI:** 10.1371/journal.pone.0180579

**Published:** 2017-07-12

**Authors:** Valentina Tosetti, Jenny Sassone, Anna L. M. Ferri, Michela Taiana, Gloria Bedini, Sara Nava, Greta Brenna, Chiara Di Resta, Davide Pareyson, Anna Maria Di Giulio, Stephana Carelli, Eugenio A. Parati, Alfredo Gorio

**Affiliations:** 1 Department of Cerebrovascular Diseases, Fondazione IRCCS Istituto Neurologico Carlo Besta, Milano, Italy; 2 Laboratory of Pharmacology, Department of Health Sciences, University of Milan, Milan, Italy; 3 Vita-Salute University and San Raffaele Scientific Institute, Division of Neuroscience, Milan, Italy; 4 Clinic of Central and Peripheral Degenerative Neuropathies Unit, Department of Clinical Neurosciences, Fondazione IRCCS Istituto Neurologico Carlo Besta, Milan, Italy; 5 Cell Therapy Production Unit, Laboratory of Cellular Neurobiology, Cerebrovascular Unit, and Unit of Molecular Neuro-Oncology, Fondazione IRCCS Istituto Neurologico Carlo Besta, Milan, Italy; 6 Biostatistician Service Clinical Research—Scientific Department, Fondazione IRCCS Istituto Neurologico Carlo Besta, Milan, Italy; 7 Vita-Salute San Raffaele University, Milan, Italy; 8 Division of Genetics and Cell Biology, IRCCS San Raffaele Scientific Institute, Milan, Italy; 9 Neurological Rare Diseases of Adulthood Unit, Department of Clinical Neurosciences, Fondazione IRCCS Istituto Neurologico Carlo Besta, Milan, Italy; 10 Pediatric Clinical Research Center Fondazione Romeo e Enrica Invernizzi, University of Milan, Milan, Italy; Universite Clermont Auvergne, FRANCE

## Abstract

The complex architecture of adult brain derives from tightly regulated migration and differentiation of precursor cells generated during embryonic neurogenesis. Changes at transcriptional level of genes that regulate migration and differentiation may lead to neurodevelopmental disorders. Androgen receptor (AR) is a transcription factor that is already expressed during early embryonic days. However, AR role in the regulation of gene expression at early embryonic stage is yet to be determinate. Long non-coding RNA (lncRNA) Sox2 overlapping transcript (Sox2OT) plays a crucial role in gene expression control during development but its transcriptional regulation is still to be clearly defined. Here, using Bicalutamide in order to pharmacologically inactivated AR, we investigated whether AR participates in the regulation of the transcription of the lncRNASox2OTat early embryonic stage. We identified a new DNA binding region upstream of Sox2 locus containing three androgen response elements (ARE), and found that AR binds such a sequence in embryonic neural stem cells and in mouse embryonic brain. Our data suggest that through this binding, AR can promote the RNA polymerase II dependent transcription of Sox2OT. Our findings also suggest that AR participates in embryonic neurogenesis through transcriptional control of the long non-coding RNA Sox2OT.

## Introduction

In the developing telencephalon, a large number of neurons originate from neuroepithelial cells and migrate across telencephalic regions. This occurs at approximately between embryonic day (E) E10.5 and E12 in mice when neuroepithelial cells transform into radial glia cells that possess neural stem cells (NSCs)/progenitor cells features [1–3]. This process is tightly regulated at transcriptional level and abnormal gene expression lead to severe neurodevelopmental disorders such as autism spectrum disorders, seizure disorders, and intellectual disability [4–6]. Thus, the understanding of molecular mechanisms that control gene expression in the earliest stages of neurogenesis (E10-E12) is crucial to elucidate the etiology of neurodevelopmental diseases.

Androgen receptor (AR) is a ligand dependent nuclear transcription factor [7] that binds with high affinity to cis-acting androgen response elements (AREs) located on nuclear chromatin adjoining androgen-responsive genes to directly regulate their transcription [8–9]. AR is suggested to have a role in the regulation of transcription during early neurogenesis because is expressed with no sex-dependent differences [10–11] both in rat embryonic neural stem cells (embryonic NSCs) and rodents adult neural stem cells (aNSCs). Recent studies on AR-mediated transcriptional programs led to the identification of molecular interactions between AR and different classes of non-coding RNAs such as PSA, HOTAIR, KLK3, PRNCR1 and PCGEM1 [12–16]. However, the identity of the AR-regulated non-coding RNAs that are critical for neurogenesis remain largely unknown.

Sox2OT is a long non-coding RNA, characterized by high degree of evolutionarily conservation [17], that acts as an enhancer during brain development, participates in transcriptional regulation of embryonic neurogenesis events [17–18] and also has a positive role in transcription regulation of SOX2 gene that is one of the major regulator of pluripotency [17–18]. The multi-exon Sox2OT has several transcription start sites (TSSs) [17,19], no open reading frame (ORF) and is spliced into several mRNA-like transcripts with the longest one of approximately 3.5 kb in human [17]. Sox2OT gene contains transposon-free non-coding regions that encompass regulatory sequences involved in the control of gene expression during early embryogenesis [17, 20].

Although Sox2OT is dynamically regulated in the mammalian embryogenesis, and it is expressed in mouse and human brain, little is known about its transcriptional regulation mechanisms.

We tested here the hypothesis that AR plays a transcriptional role in the expression of Sox2OT in early mouse neurodevelopmental stages. To block the transcriptional regulation of androgen responsive genes we used a pharmacological approach based on Bicalutamide treatment. Bicalutamide is an FDA-approved non-steroidal AR pure antagonist [21,22], that binds to cytoplasmic AR triggering its rapid degradation [23], thus it prevents AR activation and nuclear translocation and, consequently, blocks the transcriptional regulation of androgen responsive genes [23].

## Materials and methods

### Mice

Animal studies were approved by the Ethics Committee of the Carlo Besta Neurological Institute, and were conducted in accordance with the guidelines of the Italian Ministry of Health. The use and care of animals followed Italian law DL 116/1992 and EU directive 2010/63/EU. C57BL6J wild-type littermate mice (Charles River Laboratories) were used for all experiments.The animals were housed in our pathogen free facility under 12 h light/12 h dark conditions. Mice were given water and diet ad libitum. Mice were monitored daily by members of the laboratory and by animal health technicians.

### Bicalutamide treatments

Bicalutamide(N-[4-cyano-3-(trifluoromethyl)phenyl]-3-(4-fluorophenyl)sulfonyl- 2-hydroxy-2-methylpropanamide) is non-steroidal AR pure antagonist that binds to cytoplasmic AR triggering its rapid degradation[21,22]. According to the literature is more potent than other antagonist such as OH-flutamide[22,24–27]; Bicalutamide was purchased from RatiopharmTeva group. A working solution (10 mM) was created by dissolving the compound in dimethyl sulfoxide (DMSO, Sigma).

Embryonic NSCs: the optimal concentration for all in vitro experiments was 1μM for 12h supplemented after 3 days of embryonic NSCs culture. Treatments were applied daily without media change. Control cells were cultured in appropriate culture media with 0.01% of DMSO.

Mouse treatments: according to the literature, AR activation in rats embryos depends on mother testosterone serum levels [28–31]; at this stage testosterone production is mainly due to a placental steroidogenic tissue and rats embryos do not contribute to the mother secrete testosterone levels. To pharmacologically inactivate AR in mouse embryos, Bicalutamide were administered to pregnant female mice on days E10 and E11 by intraperitoneal injection at the 200μg/Kg in sterile water made up to a total volume of 200 μl. Subsequent injections of the same dose of Bicalutamide in a lower volume of DMSO did not cause toxicity. Control embryos (untreated) were obtained from pregnant female mice intraperitoneal injected at the equal volume of DMSO alone for the same time course.

### Isolation and culture of embryonic NSCs

Pregnant female were sacrificed by cervical dislocation under anesthesia at gestational E14 stage, embryos were dissected out of the amniotic sacs and the meninges were removed from the telencephalon. The forebrains were triturated and dissociated into single-cell suspensions by flushing through a p200 pipette tip and washed twice in Dulbecco's modified Eagle's medium (DMEM):F12 supplemented with 2% B27 (Invitrogen). Cells were plated at a density of 20 cells/μl in the embryonic NSCs medium: DMEM:F12 Glutamax supplemented with 2% B27 (Invitrogen), 2 μg/mL heparin and 20 ng/mL EGF and 10 ng/mL FGF-2 (Peprotec). Cultures were incubated at 37°C in a humidified atmosphere containing 5% CO2. Four days after plating, neurospheres were dissociated and subcultured as following typical neurosphere growth protocols [32]. All conditions were done in duplicate and repeated 2–4 times. Cells were re-plated at equal cell density for each condition, and numbers of neurospheres were counted after 5 days by microscopy. For ICC embryonic NSCs at 3 days of cultures were placed into 8-well chamber MATRIGEL (BectonDickinson)-coated, fixed and immunofluorescence analyses were carried out.

### RNA extraction and real-time PCR (RT-qPCR).

Total RNA was isolated from 1 × 10^6^ embryonic NSCs and E12.0 forebrains using the RNeasy microkit (Qiagen) according to the manufacturer’s instructions. cDNA was synthesized using 1 μg of total RNA and the first-strand cDNA synthesis kit (Biorad) according to the manufacturer’s instructions. Real-time PCR was performed using iTaq SYBR Green Supermix (Bio-Rad) using CFX 96 Real Time System (Bio-Rad). All real time PCR reactions were performed in triplicate.

Primer efficiencies was close to 100% for both target and reference gene. RT-qPCR was performed using CFX 96 Real Time System (Bio-Rad) and melting curve analysis was always performed at the end of each PCR assay to control specificity. The delta Ct (ΔCt) method was performed to determine relative concentrations using the average of the Ct of mouse GAPDH as normalizing value. High ΔCt values represent low levels of expression and vice versa. the primers used in RT-qPCR were: mouse GAPDH 5’-aactttggcattgtggaagg-3’ and 5’-acacattgggggtaggaaca-3’; ar 5’-ttgcaagagagctgcatcagtt-3’ and 5’-actgtgtgtggaaatagatgggc-3’; Sox2OT 5’-tgctacaagacaacaccctga-3' and 5'-ccaaagccatcaaccagatt-3' [17].

### Western blot analyses

1 × 10^6^ embryonic NSCs s and 8 E12.0 forebrains were lysed with 500 μl ice-cold RIPA buffer (50 mMTris-HCl, pH 7.4; 150 mMNaCl; 1% NP-40; 1 mM PMSF, 10 g/mL leupeptin, 10 g/mL aprotinin, 1 mM Na_3_VO_4_), and placed on ice for 10 minutes. Upon incubation on ice, samples were then centrifuged at 13000 rpm for 10 minutes at 4°C, aliquots of each sample, containing equal amount of proteins (500μg), were separated by SDS-PAGE and transferred onto PVDF membranes, were probed with antibody against rabbit anti-androgen receptor (Thermo scientific) or mouse anti-androgen receptor. To avoid blurring of specific signals resulting from a possible cross-detection of precipitating antibodies with secondary antibodies of the Western blot, we employed antibodies to rabbit or mouse IgG light-chain as secondary antibodies (Santa Cruz). Proteins of interest were visualized with the Pierce ECL Western blotting. Densitometry analysis wasdone by ImageJ software (NIH) by measuring levels of the protein of interest relative to the internal control (β-actin), as previously described [33]. The control condition was set to 1, and the y axis values show AR/ ACTIN ratio.

### Immunocytochemistry (ICC) and fluorescence in situ hybridization (FISH)

Immunocytochemistry: Cell preparations for analysis of embryonic NSCs were performed as described in Ferri et al., [34] and Favaro et al., [35]. For the anti-AR Santa Cruz (Santa Cruz Biotechnology Biotechnology) immunofluorescence, embryonic NSCs were fixed in 100% methanol for 10 minutes at -20°C. To test the specificity of the primary antibodies, negative controls (samples treated in parallel without the application of the primary antibody) were performed for each experiment. The z stacks of confocal images were taken with an optical slice thickness of 0.1 μm, with a 60× objective on the spinning disk confocal microscope (ZEISS).

#### FISH

Procedure was adapted from Schaeren-Wiemers and Gerfin-Moser [36] and Henrique et al.,[37] sense and antisense alexafluor 488-labelled RNA probes (lysis nucleic acid labeling kit—life technologies) were synthesized from non coding Sox2OTcDNA amplified sequence obtained using the following primers: 5’- tgctacaagacaacaccctga-3’ and 5’- ccaaagccatcaaccagatt-3’. The cDNAsequence was verified by sequencing. After FISH, immunostaining was performed for AR on neurospheres as describe above. Data were analyzed in 15–20 images per experiment from 3 experiments with each antibody pair.

### DNase I hypersensitivity (DHS) analysis

Approximately 2 × 10^7^ of embryonic NSCs and 10 E12.0 embryos freshly dissected mouse forebrains, were using for each DHS assay adapted from Ling and Waxman [38] and Ling et al., [39] for embryonic NSCs and brains nuclei preparation respectively. Optimization protocol provided five tubes of equally amount of chromatin incubated in parallel wherein four contained 2 units of DNase I (Sigma-Aldrich) in 100μl of the DNase I digestion buffer for 4 different incubation time (6, 8, 9, 10 minutes) at room temperature (RT) and one without enzyme (control sample) prepared in the same way by incubating in digestion buffer for 10 minutes a RT. The control DNase I digestion sample that yielded a smear of DNA fragments ranging from 100 bp to 1.5 kb was selected. digested chromatin was purified and amplifying with ARSO-Sox2OT 5’gaaatcggtggccagtgatc -3’ and 5’- ggtggacttgcttttactagagtgc -3’. For validation of DNAse digestion the following PCR primers were used:GAPDHpromoter: 5'- accagggagggctgcagtcc -3' and 5'-tcagttcggagcccacacgc -3' as a positive control (DNAse I hypersensitive region); CRISP enhancer 5'- agttcaattctctggctgatgct-3'and 5'-gaaggtgagccttatctggatagtt 3' as a negative control (DNAse I insensitive region) [40].We calculated the intensities of PCR-bands using ImageJ, verifying for non-saturation and subtracting background.

### Chromatin immunoprecipitation (ChIP) assay

ChIP was performed using approximately 2 × 10^7^ of embryonic NSCs at passages between 2–4 and 10 E12.0 forebrains for each assay (n = 1).

#### Embryonic NSCs

The pellet was resuspended in 5 ml PBS containing 1% final concentration formaldehyde and incubated for 10 min at room temperature with rotation. Cross-linking reactions were stopped with 0.125 M glycine for 8 min at room temperature and washed twice with ice-cold PBS.

#### Forebrains

Mouse embryos were harvested from timed pregnant females (Charles River) at E12.0. The forebrains were dissected in cold PBS and batches of eight forebrains each were collected in a tube (n = 1), washed twice, cut to <1mm size and crosslinked with 1% formaldehyde for 10min stopped with 0.125 M glycine for 10 min at room temperature. Chromatins from forebrain tissue and embryonic NSCs were isolated following this procedure: Nuclei were isolated and chromatin was sheared to approximately 600 bp using a sonicator (Bandeline SONOPLUS). Cross-linked chromatin was immunoprecipitated using ChIP-grade antibodies: 5 μg anti Androgen Receptor (C-19 Santa Cruz Biotechnology), 3 μg anti Phospho-Rpb1 CTD pSer2+5 (Thermo scientific) and 2 μg IgG control antibodies (Pierce) overnight at 4°C. immunoprecipitation and DNA recovery were obtained according to the manufacturer's protocol pierce magnetic chip kit (pierce). Aliquots of the purified DNA were diluted and analyzed by PCR with the following primer pairs: arso-Sox2OT 5’-ctattttcccctcgcttaacctc-3’ and 5’-tctgggtctaaagtgggcat -3’; primers for non target sequence (negative control) were 5'-attaagacacaaaggagagaggtcc-3' and 5'-tgtcatgtatcaagtttccaaaacc-3'. These non target sequence primers were previously used in ChiP-seqexperiments aimed at mapping genome-wide AR binding site in mouse caput epididymis[40]. The primers for a positive control are included in magnetic ChIP kit (Pierce). Amplification was performed for a predetermined optimal number of cycles. PCR products were separated by electrophoresis on 2% agarose gels, and stained with ethidium bromide.Relative level of chromatin was determined by quantitative densitometry using ImageJ software (NIH, Bethesda, MD). ChIP densitometry data were normalized to input (20% of total chromatin), and shown as box-plot. Fold enrichments minimum and maximum percentage are depicted by black dots, the box signifies the upper and lower quartiles, and the median is represented by a short black line within the box for each group. Validation of ARSO genome DNA sequences was performed by PCR amplification, followed by Sanger sequencing. All PCR products were sequenced in both directions using Big Dye Terminator reactions and loaded on an ABI PRISM 3730xl DNA analyzer. Sequences were analyzed using the Sequencing Analysis 5.2 software.

### RNA immunoprecipitation (RIP) assay

Approximately 1 × 10^7^ of embryonic NSCs and 10 E12.0 freshly dissected mouse forebrains was using for each RIP assay performed as previously described [41]. Total RNA was immunoprecipitated with following antibodies: 5 μgr Androgen Receptor (C-19 Santa Cruz Biotechnology), 3 μg anti Phospho-Rpb1 CTD pSer2+5 (Thermo scientific) and 2 μg rabbit IgG control antibody (Pierce) overnight at 4°C.The immunoprecipitated RNA was treated with DNase (Turbo DNase, Life Technologies, Ambion®) at 37°C for 10 min and extracted using the TRIZOL reagent (Life technologies), and reverse-transcribed using Superscript II and oligo(dT) primers, as described in the manufacturer’s protocols (BIORAD). An equal volume of RNA incubated without Superscript II was used as negative control (RT–). cDNA samples were analyzed by semiquantitative PCR with following primers: Sox2OTRNA 5’-aaaagcaagtccaccagcag -3’ and 5’-tctgggtctaaagtgggcat -3’; Amplification was performed for a predetermined optimal number of cycles (30–35). PCR products were separated by electrophoresis and stained with ethidium bromide. The PCR for Sox2OTRNA was verified using automated direct sequencing (ABI 3730, Applied Biosystems Inc., CA, USA). All PCR products were evaluated on a 2% agarose gel, sequenced in both directions using Big Dye Terminator reactions and loaded on an ABI PRISM 3730xl DNA analyzer. Sequences were analyzed using the Sequencing Analysis 5.2 software. To normalize our data the quantitative densitometry ImageJ software was used. Quantification of PCR products was performed over three independent experiments using ImageJ software (described above). All RIP densitometry results were normalized to input (20% of total RNA).

### Statistical methods

All statistical analyses were performed using GraphPad Prism 7.0 Software. Data were analyzed by Student's two-tailed, paired t-test and results were expressed as the mean value ± SEM. All experiments were carried out on a minimum of 4 occasions unless stated otherwise (n = number of independent experiments). The asterisks in each graph indicate statistically significant changes:* p < 0.05, ** p < 0.01, *** p <0.001.

## Results

### AR downregulation by Bicalutamide elicits Sox2OT downregulation

**Fig 1:**
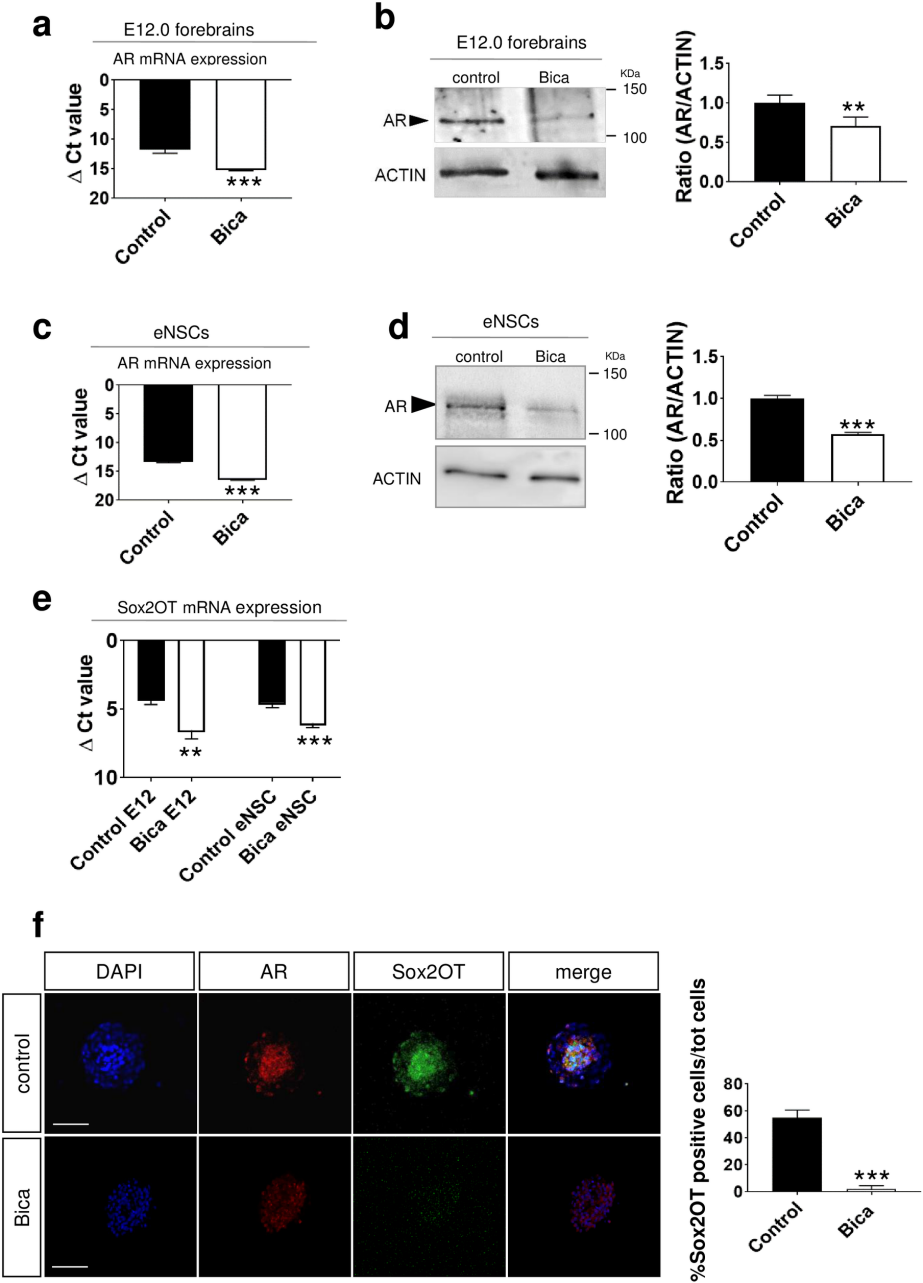
AR antagonist Bicalutamide reduces AR and Sox2OT levels in mouse E12.0.

Previous data suggested that AR mRNA in CNS is detectable at mouse embryonic day 12.5 (E12.5) shortly before embryonic hormone production [42]. To confirm that AR is expressed at early embryonic stage, we analyzed AR mRNA and protein levels in E12.0 mouse forebrains (telencephalon and diencephalon) in basal condition and after treatment with the non-steroidal AR pure antagonist Bicalutamide [21] that binds to cytoplasmic AR thus triggering its rapid degradation [23]. We found that AR mRNA is expressed in E12.0 mouse forebrains and that Bicalutamide significantly reduced AR mRNA levels (mean value±SEM of Δct (ct_AR_-ct_GAPDH_):Δct control = 11.46±0.46, ΔctBicalutamide = 14.97±0.19; t-test p = 0.0004;2-^ΔΔCt^control = 1.00, 2-^ΔΔCt^ Bicalutamide = 0.09; **Fig 1A**). To confirm this data we analyzed AR protein levels. We found detectable AR protein levels in E12.0 mouse forebrains; Bicalutamide significantly reduced AR protein levels (normalized mean value±SEM, AR/actin: control = 1.00±0.05; Bicalutamide = 0.71±0.06; t-test p = 0.0079; **Fig 1B**). Since in E12.0 forebrains embryonic neural stem cells (embryonic NSCs) are the primary progenitor cells that initiate lineages leading to the formation of differentiated neurons [43,44], we hypothesized that AR is also expressed in embryonic NSCs. To test this hypothesis we evaluated AR mRNA and protein levels in mouse embryonic NSCs in basal conditions and after treatment with Bicalutamide. Our data confirmed that AR mRNA and AR protein are expressed in embryonic NSCs (mean value±SEM ofΔct (ct_AR_-ct_GAPDH_):Δct control = 13.09±0.19; Δct Bicalutamide = 16.17±0.17, t-test p = 0.0005; 2-^ΔΔCt^ control = 1.00, 2-^ΔΔCt^ Bicalutamide = 0.12.Normalized mean value ± SEM AR/actin: control = 1.00±0.02; Bicalutamide = 0.57±0.009 t-test p = 6.4 x10^-7^;**Fig 1C and 1D).** This result agrees with previous data showing that AR is expressed in rodent adult NSCs [10, 45]. The presence of the transcription factor AR at early embryonic day and in embryonic NSCs, led us to hypothesize that AR has a role in transcriptional events regulating early neurogenesis [46,47].

Sox2OT is a lncRNA transcribed in the same orientation of Sox2 that can act as an enhancer during brain development and participate in Sox2 transcriptional regulation [17,18]. To test whether AR expression modulates Sox2OT expression we pharmacologically inhibited endogenous AR by Bicalutamide treatment in embryonic NSCs and E12.0 forebrains and analyzedSox2OT mRNA level using primers designed to probe Sox2OT last exon [17]. Bicalutamide significantly decreased Sox2OT mRNA levels (mean value ± SEM of Δct (ct_Sox2OT_-ct_GAPDH_): for E12.0:Δct control = 4.26±0.20; Δct Bicalutamide = 6.51±0.32, t-test p = 0.010; 2-^ΔΔCt^control = 1.00,2-^ΔΔCt^Bicalutamide = 0.21; for eNSCs control = 4.51±0.18; Δct Bicalutamide = 6.00±0.16, t-test p = 0.0009;2-^ΔΔCt^ control = 1.00, 2-^ΔΔCt^Bicalutamide = 0.36; **Fig 1E**). To confirm this result, we analyzed AR by immunofluorescence and Sox2OT mRNA by *in situ* hybridization in embryonic NSCs and found that Bicalutamide treatment decreased both AR and Sox2OT signals (mean value±SEM of % Sox2OT positive cells: control = 54.87±1.69, Bicalutamide = 2.19±0.70, t-test p = 5.9 x10^-11^; **Fig 1F**). Hence, these results show that AR and Sox2OT are expressed in E12.0 forebrains and in embryonic NSCs and that AR downregulation elicits Sox2OT downregulation. This result suggested that AR is directly involved in the transcriptional regulation of Sox2OT in embryonic NSCs and E12.0 forebrains.

#### Forebrains and embryonic NSCs (eNSCs)

**(a)** Real-time quantitative RT-qPCR analysis of AR mRNA in E12.0 forebrains. E12 embryos were exposed daily to Bicalutamide 200μg/Kg from E10.0 onwards. AR expression was higher in control samples than in Bicalutamide treated samples. Data are expressed as mean value ± SEM of delta ct (Δct is the Ct value for any sample normalized to the endogenous housekeeping gene): Δct control = 11.46±0.46; ΔctBicalutamide = 14.97±0.19; two-tailed t-test p = 0.0004, n = 4 samples analyzed for each condition.

**(b)** Representative western blot showing AR expression in control E12.0 forebrains and E12.0 forebrains treated with Bicalutamide. Bicalutamide significantly decreased AR protein levels. The histograms represent the mean of densitometry calculations for western blot data. Data were normalized to β-actin expression. Values are expressed as normalized mean value ± SEM of triplicate experiments: control = 1.00±0.05; Bicalutamide = 0.71±0.06; p = 0.0079 two-tailed t-test.

**(c)** AR expression in eNSCs analyzed by real-time PCR. Bicalutamide significantly decreased AR expression. Values are expressed as mean±SEM of Δct (ctAR-ctGAPDH). Δct control = 13.09±0.19, ΔctBicalutamide = 16.17±0.17; two-tailed t-test p = 0.0005; n = 5 samples for each condition.

**(d)** Representative western blot showing AR expression in control eNSCs and eNSCs treated with Bicalutamide. Data are normalized to β-actin protein. Values are expressed as normalized mean value±SEM of triplicate experiments, n = 5 samples for each condition. AR/actin: control = 1.00±0.02; Bicalutamide = 0.57±0.01, two-tailed t-test p = 6.4 x10^-7^.

**(e)** Sox2OT mRNA expressions analyzed by Real-time quantitative RT-PCR in E12.0 brains And eNSCs treated or untreated with Bicalutamide (data are expressed as mean value±SEM of Δct). For E12.0 brains: Δct control = 4.26±0.20; ΔctBicalutamide = 6.51±0.32, p = 0.010; for eNSC: control = 4.51±0.18; ΔctBicalutamide = 6.00±0.16, p = 0.0009; n = 4 samples analyzed for each condition, two-tailed t-test.

**(f)** Representative images showing AR protein signal (immunofluorescence with AR antibody in red) and Sox2OT mRNA (in situ hybridization for Sox2OT in green) in eNSCs. The histograms show the percentage of Sox2OT positive cells normalized to total nuclei counted for each neurosphere. Bicalutamide decreases both AR protein levels and the number of Sox2OT positive cells. Values are expressed as mean±SEM of %Sox2ot positive cells: control = 54.87±1.69, Bicalutamide = 2.19± 0.70, two-tailed t-test p = 5.9 x10-11; n = 5 samples analyzed for each condition. Scale bar is 50 μm.

### AR downregulation decreases the DNase hypersensitivity of ARSO-Sox2OT

**Fig 2:**
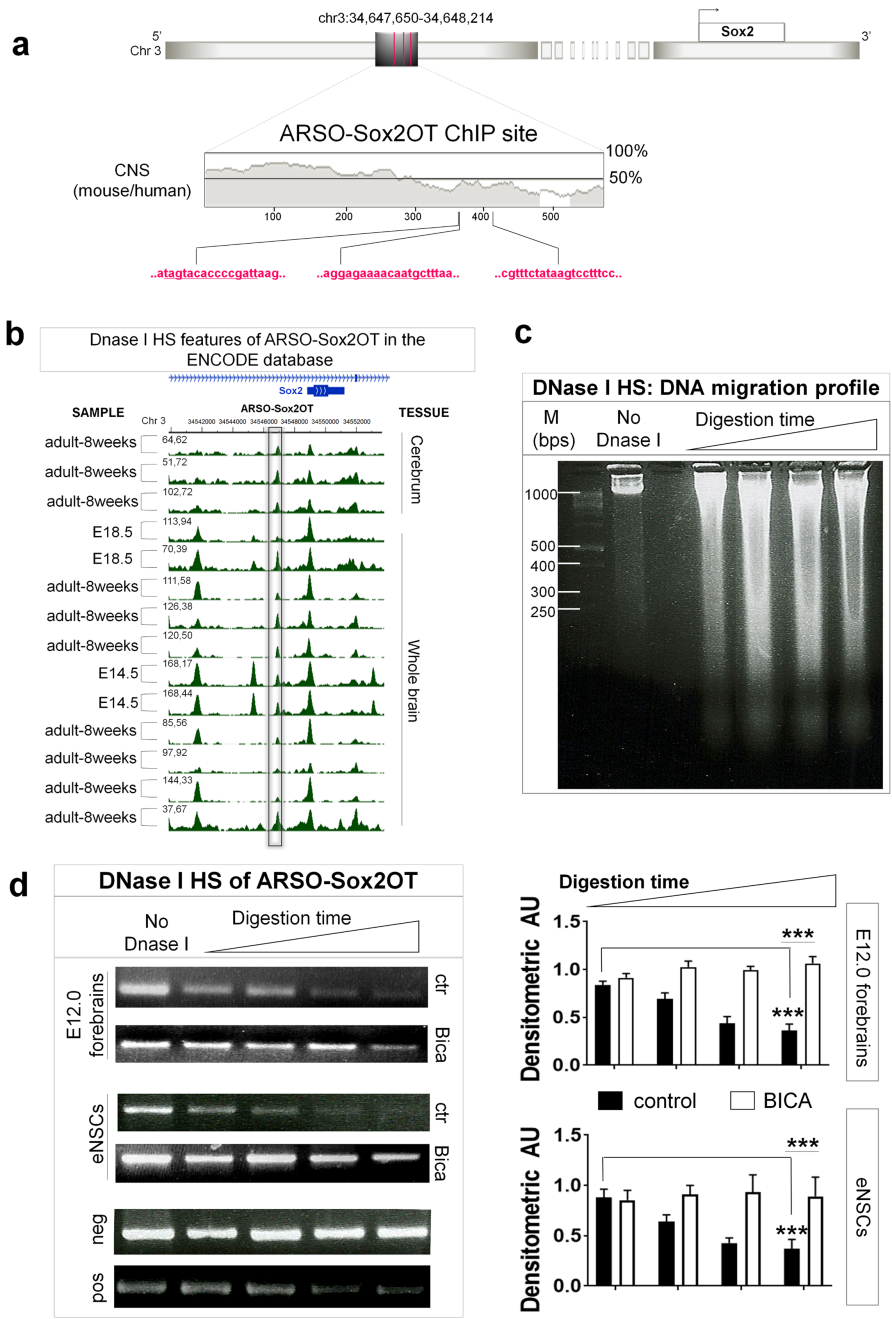
AR chromatin occupancy site (ARSO-Sox2OT) in mouse E12.0 forebrains and eNSCs. **(a)** AR consensus sites in the genomic context of SOX2 upstream region. White box, Sox2 gene; gray boxes, region tested by ChIP with ChIP-grade antibodies; vertical red lines, AR consensus site (ARE). The arrow show the direction of Sox2 gene transcription. The phylogenetic conservation (CNS) mouse-human of ARSO-Sox2OT sequence is shown below the gene diagram (derived from the VISTA browser). High sequence conservation of ARE (UCSC browser) at aligned sites across the species (shown at the left). Identical nucleotides are shown in the color pink. **(b)** Integration at Sox2 locus of ENCODE high-throughput experiments performed in mouse cerebral tissues. Rectangular heading shows theARSO-Sox2OT chromatin region. From top to bottom, row reports the genomic coverage obtained by DNAse-Seq and ChIP-Seq against different histone modifications. The image was generated using Wash U Epigenome Browser. **(c)** Right panel: Pulsed-field electrophoresis of genomic E12.0 subjected to time course digestion with DNase I (0.2 U/ml) for 6, 8, 9 and 10 minutes. **(d)** Left panel: DNA extracted from samples untreated or treated with Bicalutamidewas used as template for PCR for ARSO-Sox2OT sequence, one negative control (CRISP enhancer, neg) and one positive control (GAPDH promoter, pos). The image show representative ethidium bromide-stained gels. The data show that in control samples the ARSO-Sox2OT region is digested by DNase I whereas in samples treated with Bicalutamide the ARSO-Sox2OT region was more resistant to DNase I digestion. This result show that AR allows the chromatin accessibility of ARSO-Sox2OT region. Right panel: densitometry values of amplicones assessed by PCR for E12.0 forebrains and eNSCs. Data are expressed as mean value ± SEM of densitometry arbitrary units (AU); for E12.0 forebrains control: initial time of digestion = 0.83±0.04, final time of digestion = 0.36±0.07, t-test p = 0.00002; for E12.0 forebrains Bicalutamide: initial time of digestion = 0.91±0.04, final time of digestion = 1.06±0.07; control vs Bicalutamide at final time of digestion t-test = 0.0000075. For embryonic NSCs control: initial time of digestion = 0.87±0.08, final time of digestion = 0.37±0.09; t-test p = 0.00018; for embryonic NSCs Bicalutamide: initial time of digestion = 0.85±0.09, final time of digestion = 0.89±0.20; control vs Bicalutamide at final time of digestion t-test p = 0.0026. Results derived from five independent experiments. M: molecular size marker (base pairs, bps); No DNAse I: chromatin not digested. Ctr: no Bicalutamide treatment. Samples were treated with Bicalutamide as described in Fig 1.

The evidence showing that AR downregulation elicits Sox2OT downregulation (**Fig 1E and 1F**) led us to hypothesize that AR may interact with chromatin of Sox2OT gene. To test this hypothesis we first analyzed the presence of androgen response elements (AREs) in the 5000 bps upstream of the Sox2 translational start site (ATG) that encompass the Sox2OT locus. High density hits with a score over 0.80 and elevated conservation (over 50% of identity) of the candidate AREs sites were considered. By JASPAR motifs, we identified a sequence located -2356 bps upstream to Sox2 ATG (hereafter called ARSO-Sox2OT; chr3:34,647,650–34,648,214) containing 3 neighboring putative ARE sites including 5’-tagtacaccccgatt-3’ at site -1995, 5’-gagaaaacaatgctt-3’at position -1977 and 3’-aaggacttatagaaa-5’ at site -1933 (**Fig 2A**). Accordingly, comparative genomic analysis using the VISTA Browser (http://genome.lbl.gov/vista/index.shtml), showed high conservation among the species for ARSO-Sox2OT sequence (**Fig 2A and S1 Fig**). Data from Fig 2A suggested that ARSO-Sox2OT sequence containing the AR DNA-binding sites, is a domain of euchromatin. To further clarify aspects of the accessible chromatin landscape, we explored the epigenetic features of ARSO-Sox2OT sequence in the ENCODE Project database (http://genome.ucsc.edu/ENCODE/) focusing on whole brain, cortex and cerebrum tissues of E14.5, E18.5 and adult mice (8 weeks) (**S2 Fig**). The results obtained by ENCODE Open Chromatin by DNase-Duke University and Open Chromatin by DNase-Washington University showed that ARSO-Sox2OT is a region of chromatin highly sensitive to cleavage by DNase I (**Fig 2B**). Because DNase I hypersensitive sites (DHS) are structural landmarks indicative of regulatory chromatin regions involved in the cell-type-specific regulation [48,49], and frequently arise as a result of transcription factor binding, these results suggested that ARSO-Sox2OT is an open chromatin region [50]. Because AR downregulation elicited Sox2OT downregulation (**Fig 1E and 1F**), we hypothesized that AR modulates the DNase I hypersensitivity of ARSO-Sox2OT. To test this hypothesis, we performed a DNase hypersensitivity assays in embryonic NSCs and E12.0 forebrains in basal conditions and after Bicalutamide treatment. The size distribution of DNA short fragments released by DNase I digestion of isolated nuclei was determined by DNA electrophoresis (**Fig 2C**) followed by PCR amplification with sequence specific primers. We found that ARSO-Sox2OT chromatin region in basal condition is highly sensitive to cleavage by DNase I both in embryonic NSCs and E12.0 forebrains (**Fig 2D**). This result confirmed the data obtained from epigenetic analysis (**Fig 2B**). We also found that Bicalutamide treatment significantly decreased the hypersensitivity to DNase I (mean value ± SEM of densitometric AU; for E12.0 forebrains control: initial time of digestion = 0.83±0.04, final time of digestion = 0.36±0.07, t-test p = 0.00002; for E12.0 forebrains Bicalutamide: initial time of digestion = 0.91±0.04, final time of digestion = 1.06±0.07; control vs Bicalutamide at final time of digestion t-test = 0.0000075. For embryonic NSCs control: initial time of digestion = 0.87±0.08, final time of digestion = 0.37±0.09; t-test p = 0.00018; for embryonic NSCs Bicalutamide: initial time of digestion = 0.85±0.09, final time of digestion = 0.89±0.20; control vs Bicalutamide at final time of digestion t-test p = 0.0026. **Fig 2C**).

These results show that accessible chromatin landscape of ARSO-Sox2OT correlates with the presence of AR. Since AR binding is frequently associated with significant increase in DHS signal [51] these data also suggested that AR may bind to ARSO-Sox2OT and regulates Sox2OT expression during neural development.

### AR binds the Sox2OT gene at ARE sites and modulates RNA polymerase II-driven Sox2OT gene expression

**Fig 3:**
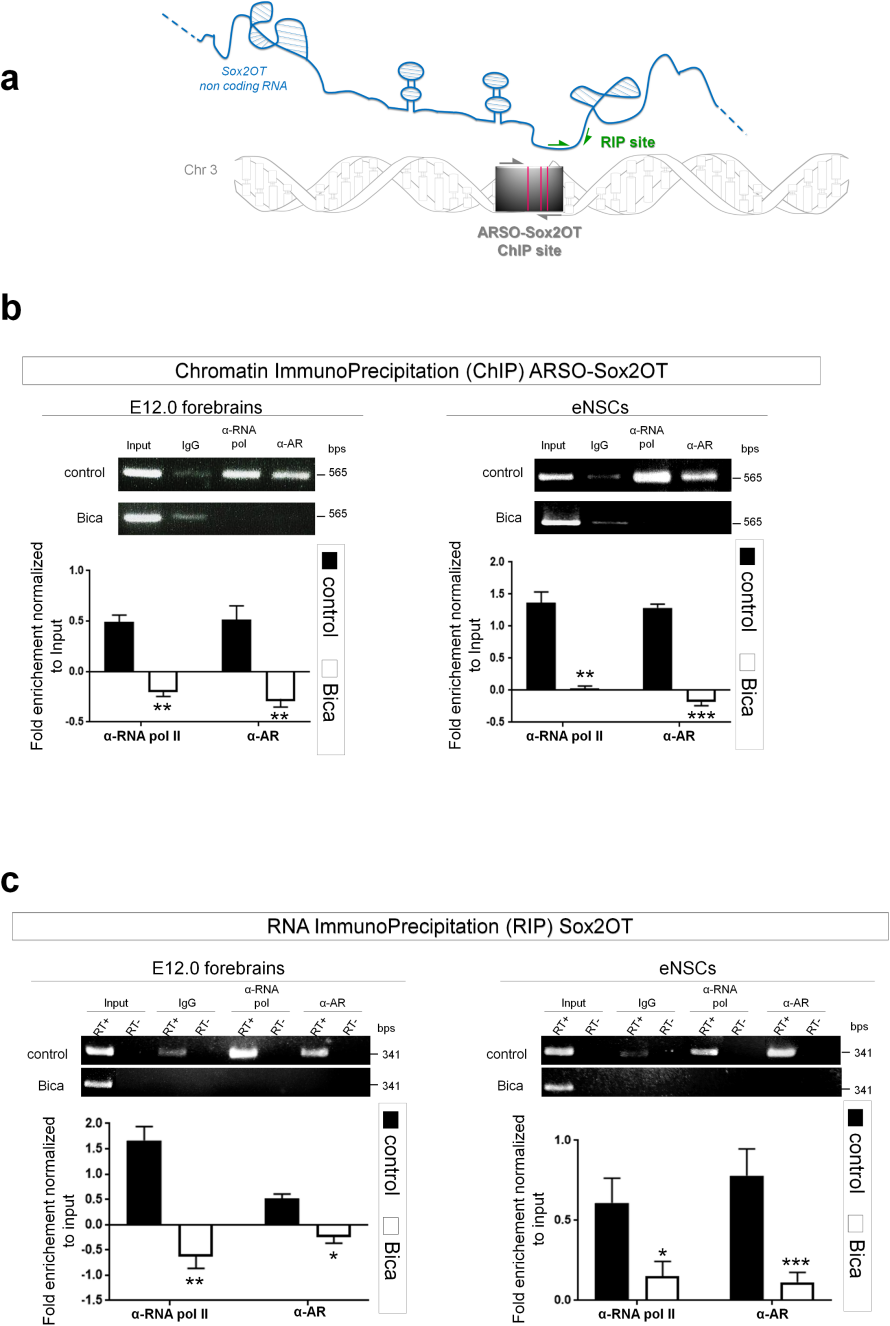
AR binds ARSO-Sox2OT chromatin region and allows Sox2OT transcription. **(a)** AR consensus sites in the chromosome 3 and in Sox2OT ncRNA. Grey arrows indicate DNA region tested by ChIP with ChIP-grade antibodies (ChIP site); vertical red lines indicate AR consensus site (ARE); green arrows indicate the sequence on Sox2OT RNA tested by RIP (RIP site); **(b)** Representative images showing Chromatin Immuno Precipitation of AR and RNA Polymerase II. The images show PCR of antibody–precipitated E12.0 forebrains and eNSCs chromatins with primers amplifying ARSO-Sox2ot region. The lower panels show the quantification of AR and RNA Pol ChIPs. Values are mean±SEM of ratios between PCR signal intensity of the AR and RNA Pol II antibodies–precipitated sample and input chromatin (IN). Notably, RNA pol II binds ARSO-Sox2ot sequence in an AR-dependent manner. For RNA pol II in E12.0 forebrains: control = 0.51±0.06, Bicalutamide = -0.18±0.06, t-test p = 0.0023. For AR in E12.0 forebrains: control = 0.51±0.13, Bicalutamide = -0.26±0.08, t-test p = 0.0003. For RNA pol II in embryonic NSCs: control = 1.36±0.21, Bicalutamide = 1.28±0.07, t-test p = 0.0002. For AR in embryonic NSCs: control = 1.28±0.07, Bicalutamide = -0.15±0.09, t-test p = 0.000017. Results derived from four independent experiments. Data were analyzed by two-tailed t-test. **(c)** RNA Immunoprecipitation (RIP) assay of eNSCs and E12.0 forebrains. RNA was subjected to IP assays with anti-AR and anti-Rpb1 CTD phosphorylated at Serine 2 and Serine 5 (RNA Pol II) antibodies or normal rabbit IgG as described in Materials and Methods section. RNA immunoprecipitates and input lysate RNAs were reverse transcription-PCR (RT-PCR)–amplified to measure the abundance of Sox2OT RNA present in the eNSCs and forebrains (control). Agarose gel of RT-PCR products from RIP and input (upper panel). Molecular weight marker sizes (base pair lengths; bps) are shown at the right. Values are mean± SEM of ratios between PCR signal intensity of the AR and RNA Pol II antibodies–precipitated sample and input (lower panel). For RNA pol II in E12.0 forebrains: control = 1.66±0.26, Bicalutamide = -0.60±0.27, t-test p = 0.0084. For AR in E12.0 forebrains: control = 0.51±0.09, Bicalutamide = -0.20±0.16, t-test p = 0,016. For RNA pol II in embryonic NSCs: control = 0.75±0.18, Bicalutamide = 0.16±0.11, t-test p = 0.032. For AR in embryonic NSCs: control = 0.85±0.07, Bicalutamide = 0.13±0.06, t-test p = 0.0002. Results are average of four independent experiments. Data were analyzed by two-tailed t-test. Lane RT+ contains an aliquot of the PCR sample. In the RT- lane, the reverse transcriptase was omitted from the RT reaction. Input are 20% of total RNA. IgG: rabbit IgG as negative control. Bicalutamide treatment were performed as described in Fig 1.

Data from Fig 2 strongly suggested that AR can bind the Sox2OT gene at the ARE sites. To test this hypothesis, we performed chromatin immunoprecipitation (ChIP) using specific ChIP-grade antibodies against AR and activated RNA polymerase II (Rbp1-CTD-p) in E12.0 forebrains and embryonic NSCs (**Fig 3A**). AR occupancy was readily detected at ARSO-Sox2OT region in concert with RNA polymerase II which transcribes the Sox2OT gene (mean value±SEM of fold enrichment normalized to input. For Rbp1-CTD-p in E12.0 forebrains: control = 0.51±0.06, Bicalutamide = -0.18±0.06, t-test p = 0.0023. For AR in E12.0 forebrains: control = 0.51±0.13, Bicalutamide = -0.26±0.08, t-test p = 0.0003. For Rbp1-CTD-p in embryonic NSCs: control = 1.36±0.21, Bicalutamide = 1.28±0.07, t-test p = 0.0002. For AR in embryonic NSCs: control = 1.28±0.07, Bicalutamide = -0.15±0.09, t-test p = 0.000017; **Fig 3B**). In tissues and cells treated with Bicalutamide neither AR nor RNA polymerase II were enriched in ChIPs (**Fig 3B)**. Treated and control templates were verified using automated direct sequencing (see material and method). These results demonstrate that AR physically binds the Sox2OT gene at new identified ARE site, suggesting that AR positively modulates the transcription of Sox2OT via recruiting and/or stabilizing the transcription complex.

To further confirm that AR mediates Sox2OT transcription, we assessed whether AR interact with Sox2OT transcript. We conducted AR- and RNA Polymerase II-directed RNA immunoprecipitation (RIP) coupled with qPCR using primer sets targeting the ARE elements at the sense Sox2OT RNA (**Fig 3A)**. We found that AR formed a ribonucleoprotein complex with active form of RNA polymerase II that was not detected in Bicalutamide treatment. These results show that AR binds Sox2OT RNA in embryonic NSCs and E12 forebrains. These data also show that the interaction between Rbp1-CTD-p and lncRNASox2OT depend on AR presence (mean value ± SEM of fold enrichment normalized to input. For Rbp1-CTD-p in E12.0 forebrains: control = 1.66±0.26, Bicalutamide = -0.60±0.27, t-test p = 0.0084. For AR in E12.0 forebrains: control = 0.51±0.09, Bicalutamide = -0.20±0.16, t-test p = 0.016. For Rbp1-CTD-p in embryonic NSCs: control = 0.75±0.18, Bicalutamide = 0.16±0.11, t-test p = 0.032. For AR in embryonic NSCs: control = 0. 85±0.07, Bicalutamide = 0.13±0.06, t-test p = 0.0002; templates treated with Bicalutamide and controls were verified using automated direct sequencing; **Fig 3C**).

## Discussion

The present study shows that AR is expressed in embryonic NSCs and E12.0 forebrains, first describe a novel androgen response element (ARE) located at Sox2OT gene and show that AR binds the Sox2OT transcript in E12.0 forebrains and embryonic NSCs.

Our evidence for AR expression in embryonic NSCs and E12.0 forebrains agrees with previous papers showing AR expression in the neurogenic territories of most vertebrate groups [52–57,45] such as rodents hippocampus, and ventricular wall of embryonic and adult rodent brains [58,45]. AR activation in rats embryos depends on mother testosterone serum levels [59–61]. In fetal life, placental and local production of sex steroids plays an important role in the expression of neuronal development genes [62,63].

To induce AR inactivation we used Bicalutamide treatment. Although this pharmacological approach have intrinsic limitations, it is an established methods to decrease AR protein and mRNA levels. Indeed, Bicalutamide triggers rapid AR degradation at a nuclear location and promotes AR turnover [64]. The mechanism of action by which Bicalutamide can decrease AR mRNA is not fully understood but evidence shows that testosterone regulates the stability of the AR mRNA by sequestering it in polyribosomes and consequently increasing its translation [65]. In this context, Bicalutamide- mediated inhibition of AR transactivational functions can lead to down regulation of its mRNA levels[66]. Alternative methods to *in vivo* inactivate AR include the in vivo delivery of small interfering RNA(siRNA) and the use of AR knock out mice. siRNAs are very efficient tools for in vitro mRNA silencing, however siRNA use in mammalian adult central nervous system is limited, because *in vivo* delivery technology such as intravenously and intraperitoneally administration of synthetic siRNAs is not fully safe and efficient. Additional important limitations exist when siRNA have to be delivered to both pregnant mice and their embryos. Regarding the possibility to perform experiments on mouse models of AR deficiency, the brain-specific models, nestinCre ARKO [67]and synapsinI Cre ARKO [68] are useful tools to study the effects of AR deficiency in adult neurons, however, in these models the conditional ablation of AR starts in the neural tube at E12.5. Thus it is difficult to assess the degree of deletion of the AR and its consequences at early stage of brain development using these models. Global AR knockout mouse (ARKO) also exist [69,70], however they are not fullycharacterized at embryonal stages. Moreover, by performing studies on mRNAs and chromatin immunoprecipitation, it is impossible to distinguish the contribution to the phenotype of loss of AR function from that resulting from the wide gene expression changes these models have.

Hence, we choose a model for pharmacological inactivation of AR using because this compound has high specificity for AR. Indeed, it was used in many studies aimed at inactivate AR [23,71,72]. Moreover, the use of Bicalutamide in the maternal system has some advantages: i) permits AR nuclear localization also in a neuronal population, ii) binds to the ligand-binding domain of the AR to inhibit its transcriptional activity [73], iii)is rapidly absorbed and it has a short plasma elimination half-life [74], iv)in our experiments, Bicalutamidewas injected intraperitoneally in the mother and no teratogenic effects were observed (data not shown).

The second novelty of our manuscript involvesthe AR binding on Sox2OT locus.AR binding to specific DNA motifs in the promoters [75–78] and in the regulatory sequences modulates many biological processes in normal and cancer cells[75–78]. Our data indicate that AR binds new identified ARE element within Sox2OT locus both in E12.0 forebrains and embryonic NSCs. ARE elements were identified by genome-wide *in silico* screening and Chip-seq analysis. These very same analyses prompted in the past to the identification of ARE sites in the genome of various human cells type [79]. At ARE sequences, AR facilitates interactions with the general transcriptional machinery leading to gene transcription of androgen responsive genes [80]. We show here that ARSO-Sox2OT sequence contains three ARE sites located in the intron of Sox2OT [17] and that AR promotes Sox2OT transcription. This result is strongly supported by evidence showing that Bicalutamide treatment decreases Sox2OT transcription. Our results also suggest that the underlying molecular mechanism involves the interaction between RNA polymerase II and AR. Previous study showed that RNA polymerase II (Pol II) binds to a large number of intergenic AR-bound enhancers marked by histone H3 lysine 4 monomethylation (H3K4me1) and lysine 27 acetylation (H3K27ac) to produce enhancer-derived long non-coding RNAs (eRNAs) [81–84]. We can therefore speculate that AR mediates the expression of Sox2OT through interacting with Pol II [85]. This hypothesis is supported by evidence showing that AR interacts with nuclear ribonucleoprotein particles from target tissues [86] and that AR activation regulates the abundance of specific RNA sequences in rat prostate, mouse liver and kidney [87–89].

Our RIP results show that AR interacts with Sox2OT mRNA. This data agrees with previous studies demonstrating that AR regulates non-coding RNAs by means of mechanisms common to protein-coding transcripts. Sheflin and colleagues [90] demonstrated that AR can act post-transcriptionally toregulate the 3'UTRs of mammalian HIF 1 alpha and EGF mRNA. Moreover a fraction of long unspliced intronic RNAs may have a role in post-transcriptional regulation of gene expression by modulating transcript stability and alternative splicing [14]. AR might function as the detachment of nascent RNA from DNA template to process Sox2OT precursor transcript [91]. Our findings represent the first evidence that AR participates in the control of Sox2OT transcript forming.

Our findings support the hypothesis that AR has a pivotal role in controlling the transcription of Sox2OT during mouse early neurodevelopmental stages. The physiological significance of the regulation of Sox2OT by AR remains open. AR-regulated Sox2OT in NSCs may regulate stemness, cell proliferation, differentiation, cell fate (into neurons or glia).

Because Sox2OT is transcribed in the same orientation of Sox2, Sox2OTcan act as an enhancer during brain development and participates in Sox2 transcriptional regulation [17,18], we can speculate that AR may play a role in the regulation of SoxB1 transcription factors including Sox2. Because Sox2, that is expressed in NSCs, it is not only involved in neurogenesis but also in gliogenesis [92] we can postulate that this pathway can modulate differentiation of neural progenitor cells versus neuronal or glial phenotypes. Finally, through shedding light on a physiological AR role in mouse embryos, our report suggests that AR-Sox2OT pathway may have a role in neurodevelopmental diseases and provide the basis for future studies aimed at clarifying the autocrine or paracrine effects of sex steroids on AR expression and function during neurodevelopment.

## Supporting information

S1 Fig AR consensus sites in the genomic context of Sox2 upstream region. White box, Sox2 gene; gray boxes, region tested by ChIP with ChIP-grade antibodies; vertical red lines, AR consensus sites (ARE). The direction of Sox2 gene transcription is shown with arrow. The phylogenetic conservation (CNS) Mouse-human of ARSO-Sox2OTsequence is shown below the gene diagram (derived from the VISTA browser). High sequence conservation of ARE (UCSC browser) at aligned sites across the species (shown at the left). Identical nucleotides are shown in the color pink.
(TIF)

S2 Fig Epigenetic features of ARSO-Sox2OT sequence. Integration at ARSO-Sox2OT site of ENCODE high-throughput experiments performed in mouse cerebral tissues. Respect to Sox2 locus at top, vertical rectangular shows the ARSO-Sox2OTchromatin region. From top to bottom, row reports the genomic coverage obtained by DNAse-Seq and ChIP-Seq against different histone modifications. Letters on the right column indicate the tissues analyzed, row by row. Sample information isreported at bottom. Top panel shows results obtained by ENCODE Open Chromatin by DNase-Duke University and Open Chromatin by DNase-Washington University. Is evident a peak in the ARSO-Sox2OT sequence, which is more present in embryonic than adult tissues.H3K4me3/1, H3K36me3, and H3K27ac are modifications associated with active chromatin and H3K27me3 and H3K9me3 for silenced heterochromatic region. The image was generated using Wash U Epigenome Browser.
(TIF)

## Abbreviations

AR: Androgen Receptor
Sox2OT: Sox2 overlapping transcript
Bica: Bicalutamide
E: embryonic day
NSCs: neural stem cells
AREs: androgen response elements
ARSO-Sox2OT: the sequence located -2356 bps upstream to Sox2 ATG
TSSs: transcription start sites
ORF: open reading frame
embryonic NSCs: embryonic neural stem cells
RIP: RNA Immuno Precipitation
ChIP: Chromatin Immuno Precipitation
ncRNAs: non-coding RNAs
lncRNA: long non-coding RNA
RT-qPCR: Real Time quantitative PCR
RNA Pol II: Rpb1 CTD phosphorylated at Serine 2 and Serine 5

## References

[1] Haubensak W, Attardo A, Denk W, Huttner WB. Neurons arise in the basal neuroepithelium of the early mammalian telencephalon: a major site of neurogenesis. PNAS., 2004;101,3196–3201. doi: 10.1073/pnas.0308600100 14963232 10.1073/pnas.0308600100 PMC365766

[2] Noctor SC, Martínez-Cerdeño V, Ivic L, Kriegstein AR. Cortical neurons arise in symmetric and asymmetric division zones and migrate through specific phases. Nat Neurosci. 2004;7, 136–144. doi: 10.1038/nn1172 14703572 10.1038/nn1172

[3] Miyata T, Kawaguchi D, Kawaguchi A, Gotoh. Mechanisms that regulate the number of neurons during mouse neocortical development. CurrOpinNeurobiol.2010; 20, 22–28. 10.1016/j.conb.2010.01.001 20138502

[4] Sugathan A, Biagioli M, Golzio C, Erdin S, Blumenthal I, Manavalan P, et al CHD8 regulates neurodevelopmental pathways associated with autism spectrum disorder in neural progenitors. ProcNatlAcadSci U S A. 2014;11, E4468–E4477. 10.1073/pnas.1405266111 PMC4210312 25294932

[5] Chuang HC, Huang TN, Hsueh YP. T-Brain-1—A Potential Master Regulator in Autism Spectrum Disorders. AutismRes. 20158, 412–426. 10.1002/aur.1456 25600067

[6] Rangasamy S, D’Mello SR, Narayanan V. Epigenetics, Autism Spectrum, and Neurodevelopmental Disorders. Neurotherapeutics.2013;10, 742–756. doi: 10.1007/s13311-013-0227-0 24104594 10.1007/s13311-013-0227-0 PMC3805864

[7] Gao W., Bohl C.E. and Dalton J.T. Chemistry and structural biology of androgen receptor. Chemical Reviews. 2005; 105(9), pp.3352–3370. doi: 10.1021/cr020456u 16159155 10.1021/cr020456u PMC2096617

[8] Heinlein C.A. and Chang C. Androgen receptor (AR) coregulators: An overview. Endocrine Reviews. 2002; 23(2), pp.175–200. doi: 10.1210/edrv.23.2.0460 11943742 10.1210/edrv.23.2.0460

[9] Wang Q, Li W, Liu XS, Carroll JS, Jänne OA, Keeton EK et al A Hierarchical Network of Transcription Factors Governs Androgen Receptor-Dependent Prostate Cancer Growth. Molecular Cell. 2007; 27(3), pp.380–392. doi: 10.1016/j.molcel.2007.05.041 17679089 10.1016/j.molcel.2007.05.041 PMC3947890

[10] Ransome MI and Boon WC. Testosterone-induced adult neurosphere growth is mediated by sexually-dimorphic aromatase expression. Front Cell Neurosci.2015;9, 253 doi: 10.3389/fncel.2015.00253 26217181 10.3389/fncel.2015.00253 PMC4491627

[11] Crocoll A, Zhu CQC, Cato ACB, Blum M. Expression of androgen receptor mRNA during mouse embryogenesis. Mechanisms of Development. 1998; 72(1–2#3625), pp.175–178. 9533962 10.1016/s0925-4773(98)00007-0

[12] Zhao Y, Wang L, Ren S, Wang L, Blackburn PR, McNulty MS, et al Activation of P-TEFb by Androgen Receptor-Regulated Enhancer RNAs in Castration-Resistant Prostate Cancer. Cell Rep. 2016;15, 599–610. doi: 10.1016/j.celrep.2016.03.038 27068475 10.1016/j.celrep.2016.03.038 PMC5395199

[13] Zhang A, Zhao JC, Kim J, Fong KW, Yang YA, et al LncRNA HOTAIR Enhances the Androgen-Receptor-Mediated Transcriptional Program and Drives Castration-Resistant Prostate Cancer. Cell Rep. 2015;13, 209–221. doi: 10.1016/j.celrep.2015.08.069 26411689 10.1016/j.celrep.2015.08.069 PMC4757469

[14] Nakaya HI, Amaral PP, Louro R, Lopes A, Fachel AA, Moreira YB, et al Androgen responsive intronic non-coding RNAs. BMC Biol.2007;5, 4 doi: 10.1186/1741-7007-5-4 17263875 10.1186/1741-7007-5-4 PMC1800835

[15] Prensner JR, Sahu A, Iyer MK, Malik R, Chandler B, Asangani IA, et al The IncRNAs PCGEM1 and PRNCR1 are not implicated in castration resistant prostate cancer. Oncotarget. 2014; 5, 1434–1438. doi: 10.18632/oncotarget.1846 24727738 10.18632/oncotarget.1846 PMC4039221

[16] Hsieh CL, Fei T, Chen Y, Li T, Gao Y, Wang X, et al Enhancer RNAs participate in androgen receptor-driven looping that selectively enhances gene activation. ProcNatlAcadSci U S A. 2014;111, 7319–7324. 10.1073/pnas.1324151111 PMC4034202 24778216

[17] Amaral PP, Neyt C, Wilkins SJ, Askarian-Amiri ME, Sunkin SM, Perkins AC, et al Complex architecture and regulated expression of the Sox2OT locus during vertebrate development. RNA.2009 15, 2013–2027. doi: 10.1261/rna.1705309 19767420 10.1261/rna.1705309 PMC2764477

[18] Askarian-Amiri ME, Seyfoddin V, Smart CE, Wang J, Kim JE, Hansji H, et al Emerging role of long non-coding RNA SOX2OT in SOX2 regulation in breast cancer. PLoSOne.2014;9, 1–10. 10.1371/journal.pone.0102140 PMC4090206 25006803

[19] Mikkelsen TS, Ku M, Jaffe DB, Issac B, Lieberman E, Giannoukos G, et al Genome-wide maps of chromatin state in pluripotent and lineage-committed cells. Nature.2007;448, 553–560. doi: 10.1038/nature06008 17603471 10.1038/nature06008 PMC2921165

[20] Simons C, Pheasant M, Makunin I V., Mattick JS. Transposon-free regions in mammalian genomes. Genome Res.2006;16, 164–172. doi: 10.1101/gr.4624306 16365385 10.1101/gr.4624306 PMC1361711

[21] Furr BJA.The development of Casodex (Bicalutamide): preclinical studies. European Urology; 1996; 29(suppl 2), pp.83–95. 8717469 10.1159/000473846

[22] Furr BJA and Tucker H.The preclinical development of Bicalutamide: Pharmacodynamics and mechanism of action. Urology; 1995pp. 13–25. 8560673 10.1016/s0090-4295(96)80003-3

[23] Masiello D, Cheng S, Bubley GJ, Lu ML, Balk SP. Bicalutamide functions as an androgen receptor antagonist by assembly of a transcriptionally inactive receptor. Journal of Biological Chemistry 2002; 277(29), pp.26321–26326. doi: 10.1074/jbc.M203310200 12015321 10.1074/jbc.M203310200

[24] Altwein JE, Schmitz-Drager B, Wirth M. Molecular Biology of Prostate Cancer. De gruyter 2013.

[25] Teutsch G, Goubet F, Battmann T, Bonfils A, Bouchoux F, Cerede E, et al J Steroid Biochem Mol Biol.1994;,48:111 8136296 10.1016/0960-0760(94)90257-7

[26] Luo S, Martel C, Chen C, Labrie C, Candas B, Singh SM, et al Daily dosing with flutamide or Casodex exerts maximal antiandrogenic activity.Urology. 1997;Dec;50(6):913–9. 9426723 10.1016/s0090-4295(97)00393-2

[27] Ayub M, Levell MJ.The effect of ketoconazole related imidazole drugs and antiandrogens on [3H] R 1881 binding to the prostatic androgen receptor and [3H]5 alpha-dihydrotestosterone and [3H]cortisol binding to plasma proteins.J Steroid Biochem. 1989;8;33(2):251–5. 2788775 10.1016/0022-4731(89)90301-4

[28] Sridaran R, Gibori G. Intraovarian localization of luteinizing hormone/human chorionic gonadotropin stimulation of testosterone and estradiol synthesis in the pregnant rat. Endocrinology. 1993;5;112(5):1770–6. 10.1210/endo-112-5-1770 6832068

[29] Warren DW, Haltmeyer GC, Eik-Nes KB. Testosterone in the fetal rat testis. Biol.Reprod.1973;8, 560–565. 4713164 10.1093/biolreprod/8.5.560

[30] Noumura T, Weisz J, Lloyd CW. Invitro conversion of 7ı3 H-progesterone to androgen by the rat testis during the second half offetal life. Endocrinology. 1966;78, 245–25 3. doi: 10.1210/endo-78-2-245 4379314 10.1210/endo-78-2-245

[31] Feldman SC Bloch E. Developmental pattern of testosterone synthesis by fetal rat testes in response to luteinizinghormone. Endocrinology. 1978;102, 999–1007. doi: 10.1210/endo-102-4-999 744029 10.1210/endo-102-4-999

[32] Fasano CA, Dimos JT, Ivanova NB, Lowry N, Lemischka IR, Temple S.shRNA Knockdown of Bmi-1 Reveals a Critical Role for p21-Rb Pathway in NSC Self-Renewal during Development. Cell Stem Cell. 2007; 1, 87–99. doi: 10.1016/j.stem.2007.04.001 18371338 10.1016/j.stem.2007.04.001

[33] Williams A, Sarkar S, Cuddon P, Ttofi EK, Saiki S, Siddiqi FH et al Novel targets for Huntington’s disease in an mTOR-independent autophagy pathway. NatChemBiol.2008; 4, 295–305. 10.1038/nchembio.79 PMC2635566 18391949

[34] Ferri AL, Cavallaro M, Braida D, Di Cristofano A, Canta A, Vezzani A, et al Sox2 deficiency causes neurodegeneration and impaired neurogenesis in the adult mouse brain. Development. 2004; 131, 3805–3819. doi: 10.1242/dev.01204 15240551 10.1242/dev.01204

[35] Favaro R, Valotta M, Ferri AL, Latorre E, Mariani J, Giachino C, et al Hippocampal development and neural stem cell maintenance require Sox2-dependent regulation of Shh. Nat Neurosci.2009;12, 1248–1256. doi: 10.1038/nn.2397 19734891 10.1038/nn.2397

[36] Schaeren-Wiemers N. and Gerfin-Moser A. A single protocol to detect transcripts of various types and expression levels in neural tissue and cultured cells: in situ hybridization using digoxigenin-labelled cRNA probes. Histochemistry. 1993; 100(6), pp.431–440. 7512949 10.1007/BF00267823

[37] Henrique D, Adam J, Myat A, Chitnis A, Lewis J, Ish-Horowicz D. Expression of a Delta homologue in prospective neurons in the chick. Nature.1995; 375(6534),787–790. doi: 10.1038/375787a0 7596411 10.1038/375787a0

[38] Ling G. and Waxman D.J. DNase I digestion of isolated nulcei for genome-wide mapping of DNase hypersensitivity sites in chromatin. Methods in Molecular Biology. 2013; 977, pp.21–33. doi: 10.1007/978-1-62703-284-1_3 23436351 10.1007/978-1-62703-284-1_3 PMC3889470

[39] Ling G, Sugathan A, Mazor T, Fraenkel E, Waxman DJ. Unbiased, genome-wide in vivo mapping of transcriptional regulatory elements reveals sex differences in chromatin structure associated with sex-specific liver gene expression. Mol Cell Biol.2010;30,5531–5544. doi: 10.1128/MCB.00601-10 20876297 10.1128/MCB.00601-10 PMC2976433

[40] Hu S, Yao G, Guan X, Ni Z, Ma W, Wilson EM, et al Research resource: Genome-wide mapping of in vivo androgen receptor binding sites in mouse epididymis. MolEndocrinol.2010;24, 2392–2405. 10.1210/me.2010-0226 PMC2999474 20943813

[41] Latorre E, Tebaldi T, Viero G, Spartà A, Quattrone A, Provenzani A. Downregulation of HuR as a new mechanism of doxorubicin resistance in breast cancer cells. Mol Cancer.2012;11, 13 doi: 10.1186/1476-4598-11-13 22436134 10.1186/1476-4598-11-13 PMC3325864

[42] Ikeda Y., Shen WH, Ingraham HA, Parker KL. Developmental expression of mouse steroidogenic factor-1, an essential regulator of the steroid hydroxylases. Molecular endocrinology (Baltimore, Md.).1994;8(5), pp.654–662. 10.1210/mend.8.5.8058073 8058073

[43] Gage F.H. Mammalian neural stem cells. Science. 2000;287(5457), pp.1433–1438. 10688783 10.1126/science.287.5457.1433

[44] Gage FH, Temple S. Neural stem cells: Generating and regenerating the brain. Neuron.2013;80, 588–601. doi: 10.1016/j.neuron.2013.10.037 24183012 10.1016/j.neuron.2013.10.037

[45] Brännvall K, Bogdanovic N, Korhonen L, Lindholm D. 19-Nortestosterone influences neural stem cell proliferation and neurogenesis in the rat brain. European Journal of Neuroscience. 2005;21(4), pp.871–878. doi: 10.1111/j.1460-9568.2005.03942.x 15787693 10.1111/j.1460-9568.2005.03942.x

[46] Juntti SA, Coats JK, Shah NM. A genetic approach to dissect sexually dimorphic behaviors. HormBehav. 2008;53, 627–637. 10.1016/j.yhbeh.2007.12.012 PMC2464277 18313055

[47] Juntti SA, Tollkuhn J, Wu MV, Fraser EJ, Soderborg T, Tan S, et al The androgen receptor governs the execution, but not programming, of male sexual and territorial behaviors. Neuron.2010; 66, 260–272. doi: 10.1016/j.neuron.2010.03.024 20435002 10.1016/j.neuron.2010.03.024 PMC2923659

[48] Reinke H. and Hörz W. Anatomy of a hypersensitive site. Biochimica et BiophysicaActa—Gene Structure and Expression. 2004;1677(1–3), pp.24–29. 10.1016/j.bbaexp.2003.09.014 15020042

[49] De Laat W. and Grosveld F. Spatial organization of gene expression: The active chromatin hub. Chromosome Research.2003;11(5), pp.447–459. 12971721 10.1023/a:1024922626726

[50] Cockerill PN. Structure and function of active chromatin and DNaseIhypersensitive sites.FEBS J. 2011;278, 2182–2210. doi: 10.1111/j.1742-4658.2011.08128.x 21501387 10.1111/j.1742-4658.2011.08128.x

[51] He HH, Meyer CA, Chen MW, Jordan VC, Brown M, Liu XS. Differential DNase I hypersensitivity reveals factor-dependent chromatin dynamics. Genome Res.2012;22, 1015–1025. doi: 10.1101/gr.133280.111 22508765 10.1101/gr.133280.111 PMC3371710

[52] Forlano PM, Marchaterre M, Deitcher DL, Bass AH. Distribution of androgen receptor mrna expression in vocal, auditory, and neuroendocrine circuits in a teleost fish. J Comp Neurol.2010;518,493–512. doi: 10.1002/cne.22233 20020540 10.1002/cne.22233 PMC2976675

[53] Kim YH, Perlman WR, Arnold AP. Expression of androgen receptor mRNA in zebra finch song system: developmental regulation by estrogen. J Comp Neurol.2004;469, 535–547. doi: 10.1002/cne.11033 14755534 10.1002/cne.11033

[54] Wood CE, Keller-Wood M. Ontogeny of androgen receptor expression in the ovine fetal central nervous system and pituitary. Neurosci Lett.2008; 432, 153–156. 10.1016/j.neulet.2008.05.008 PMC2507723 18514409

[55] Sheppard KM, Padmanabhan V, Coolen LM, Lehman MN.Prenatal Programming by Testosterone of Hypothalamic Metabolic Control Neurones in the Ewe. J Neuroendocrinol.2011;23, 401–411. doi: 10.1111/j.1365-2826.2011.02126.x 21418339 10.1111/j.1365-2826.2011.02126.x PMC3939689

[56] Gorelick DA, Watson W, Halpern ME. Androgen receptor gene expression in the developing and adult zebrafish brain. Dev Dyn. 2008; 273, 2987–2995. 10.1002/dvdy.21700 18816841

[57] Godsave SF, Lohmann R, Vloet RPM, Gahr M. Androgen receptors in the embryonic zebra finch hindbrain suggest a function for maternal androgens in perihatching survival. J Comp Neurol.2002;453, 57–70. doi: 10.1002/cne.10391 12357432 10.1002/cne.10391

[58] Raskin K, de Gendt K, Duittoz A, Liere P, Verhoeven G, Tronche F, et al Conditional inactivation of androgen receptor gene in the nervous system: effects on male behavioral and neuroendocrine responses. J Neurosci.2009;29, 4461–4470. doi: 10.1523/JNEUROSCI.0296-09.2009 19357272 10.1523/JNEUROSCI.0296-09.2009 PMC6665718

[59] Noumura T., Weisz J. and Lloyd C. W. In vitro conversion of 7ı3 H-progesterone to androgen by the rat testis during the second half of fetal life. Endocrinology.1966; 78 (2): 245–253. doi: 10.1210/endo-78-2-245 4379314 10.1210/endo-78-2-245

[60] Warren D. W., Haltmeyer G. C. and Eik-Nes K. B. Testosterone in the fetal rat testis. Biol.Reprod.1973;8, 560–565. 4713164 10.1093/biolreprod/8.5.560

[61] Feldman S. C. and Bloch E. Developmental pattern of testosterone synthesis by fetal rat testes in response to luteinizing hormone. Endocrinology.1978;102, 999–1007. doi: 10.1210/endo-102-4-999 744029 10.1210/endo-102-4-999

[62] Lombardo MV, Ashwin E, Auyeung B, Chakrabarti B, Lai MC, Taylor K, et al Fetal testosterone influences sexually dimorphic gray matter in the human brain. J Neurosci.2012;32, 674–680. doi: 10.1523/JNEUROSCI.4389-11.2012 22238103 10.1523/JNEUROSCI.4389-11.2012 PMC3306238

[63] Ronen D. and Benvenisty N. Sex-dependent gene expression in human pluripotent stem cells. Cell Rep.2014;8, 923–932. doi: 10.1016/j.celrep.2014.07.013 25127145 10.1016/j.celrep.2014.07.013

[64] Waller S, Sharrard RM, Berthon P, Maitland NJ. Androgen receptor localisation and turnover in humanprostate epithelium treated with the antiandrogen, Casodex. A Journal of Molecular Endocrinology. 2000;24, 339–351. 10828827 10.1677/jme.0.0240339

[65] Mora GR, Mahesh VB. Autoregulation of the androgen receptor at the translational level: testosterone induces accumulation of androgen receptor mRNA in the rat ventral prostate polyribosomes. Steroids. 1999;9;64(9):587–91. 10503713 10.1016/s0039-128x(99)00037-9

[66] Furutani T, Watanabe T, Tanimoto K, Hashimoto T, Koutoku H, Kudoh M, et al Stabilization of androgen receptor protein is induced by agonist, not by antagonists.Biochem Biophys Res Commun.2002; 6 21;294(4):779–84. doi: 10.1016/S0006-291X(02)00564-8 12061774 10.1016/S0006-291X(02)00564-8

[67] Juntti SA, Tollkuhn J, Wu MV, Fraser EJ, Soderborg T, Tan S, et al The androgen receptor governs the execution, but notprogramming, of male sexual and territorial behaviours.Neuron.2010; 4 29; 66(2): 260–272. doi: 10.1016/j.neuron.2010.03.024 20435002 10.1016/j.neuron.2010.03.024 PMC2923659

[68] Yu IC, Lin HY, Liu NC, Sparks JD, Yeh S, Fang LY, et al Neuronal androgen receptor regulatesinsulin sensitivity via suppression of hypothalamic NF‑kappaB‑Mediated PTP1Bexpression. Diabetes. 2013;62: 411–23. doi: 10.2337/db12-0135 23139353 10.2337/db12-0135 PMC3554386

[69] Holdcraft RW, Braun RE. Androgen receptor function is required in Sertoli cells for the terminal differentiation of haploid spermatids. Development. 2004;131: 459–67. doi: 10.1242/dev.00957 14701682 10.1242/dev.00957

[70] MacLean HE, Chiu WM, Ma C, McManus JF, Davey RA, Cameron R, et al A floxed allele ofthe androgen receptor gene causes hyperandrogenization in male mice. Physiol Genomics. 2008;33: 133–7. doi: 10.1152/physiolgenomics.00260.2007 18171720 10.1152/physiolgenomics.00260.2007

[71] Gravina G.L., Festuccia C, Millimaggi D, Tombolini V, Dolo V, Vicentini C, et al Bicalutamide Demonstrates Biologic Effectiveness in Prostate Cancer Cell Lines and Tumor Primary Cultures Irrespective of Her2/neu Expression Levels. Urology. 2009; 74(2), pp.452–457. doi: 10.1016/j.urology.2009.01.018 19285710 10.1016/j.urology.2009.01.018

[72] Giorgetti E, Rusmini P, Crippa V, Cristofani R, Boncoraglio A, Cicardi ME, et al Synergic prodegradative activity of Bicalutamide and trehalose on the mutant androgen receptor responsible for spinal and bulbar muscular atrophy. Hum Mol Genet. 2015; 1 1; 24(1): 64–75. doi: 10.1093/hmg/ddu419 25122660 10.1093/hmg/ddu419 PMC4262493

[73] Hodgson MC, Astapova I, Hollenberg AN, Balk SP. Activity of androgen receptor antagonist Bicalutamide in prostate cancer cells is independent of NCoR and SMRT corepressors. Cancer Res.2007;9 1;67(17):8388–95. doi: 10.1158/0008-5472.CAN-07-0617 17804755 10.1158/0008-5472.CAN-07-0617

[74] Cockshott ID.Bicalutamide: clinical pharmacokinetics andmetabolism.ClinPharmacokinet. 2004;43(13):855–78. 10.2165/00003088-200443130-00003 15509184

[75] Claessens F, Celis L, De Vos P, Heyns W, Verhoeven G, Peeters B, et al Functional androgen response elements in the genes coding for prostatic binding protein. Ann N Y AcadSci. 1993; 684: 199–201. 10.1111/j.1749-6632.1993.tb32283.x 8317830

[76] Cleutjens K B, Van derKorput HA, Ehren-van Eekelen CC, Sikes RA, Fasciana C, Chung LW, et al A 6-kb promoter fragment mimics in transgenic mice the prostate-specific and androgen-regulated expression of the endogenous prostate-specific antigen gene in humans. Mol Endocrinol. 1997a;11:1256–1265. 9259317 10.1210/mend.11.9.9974

[77] Schuur ER, Henderson GA, Kmetec LA, Miller J D, Lamparski HG, Henderson DR J. Prostate-specific antigen expression is regulated by an upstream enhancer.Biol. Chem.1996;271, 7043–7051. 10.1074/jbc.271.12.7043 8636136

[78] Huang W, Shostak Y, Tarr P, Sawyers C, Carey M.Cooperative assembly of androgen receptor into a nucleoprotein complex that regulates the prostate-specific antigen enhancer.M. J. Biol. Chem.1999;274, 25756–25768. 10.1074/jbc.274.36.25756 10464314

[79] Perets R, Kaplan T, Stein I, Hidas G, Tayeb S, Avraham E, et al Genome-Wide Analysis of Androgen Receptor Targets Reveals COUP-TF1 as a Novel Player in Human Prostate Cancer. PLoSOne.2012;7, e46467. 10.1371/journal.pone.0046467 PMC3464259 23056316

[80] Lee D.K. and Chang C. Molecular communication between androgen receptor and general transcription machinery. Journal of Steroid Biochemistry and Molecular Biology. 2003;84(1), pp.41–49. 12648523 10.1016/s0960-0760(03)00005-0

[81] De Santa F, Barozzi I, Mietton F, Ghisletti S, Polletti S, Tusi BK, et al A large fraction of extragenic RNA Pol II transcription sites overlap enhancers. PLoS Biol. 2010; 11,8, e1000384. 10.1371/journal.pbio.1000384 PMC2867938 20485488

[82] Kim TK, Hemberg M, Gray JM, Costa AM, Bear DM, Wu J, et al Widespread transcription at neuronal activity-regulated enhancers. Nature.2010;465(7295), 182–187. doi: 10.1038/nature09033 20393465 10.1038/nature09033 PMC3020079

[83] Wang D, Garcia-Bassets I, Benner C, Li W, Su X, Zhou Y, et al Reprogramming transcription by distinct classes of enhancers functionally defined by eRNA. Nature.2011;474(7351), 390–394. doi: 10.1038/nature10006 21572438 10.1038/nature10006 PMC3117022

[84] Ørom UA, Derrien T, Beringer M, Gumireddy K, Gardini A, Bussotti G, et al Long non coding RNAs usher in a new era in the biology of enhancers. Cell.2013;143,46–58.

[85] Louie MC, Yang HQ, Ma A-H, Xu W, Zou JX, Kung HJ, et al Androgen-induced recruitment of RNA polymerase II to a nuclear receptor-p160 coactivator complex. ProcNatlAcadSci U S A.2003;100,2226–2230. 10.1073/pnas.0437824100 PMC151322 12589022

[86] Yeap BB, Voon DC, Vivian JP, McCulloch RK, Thomson AM, Giles KM, et al Novel binding of HuR and poly(C) -binding protein to a conserved UC-rich motif within the 3’-untranslated region of the androgen receptor messenger RNA. J Biol Chem.2002;277, 27183–27192. doi: 10.1074/jbc.M202883200 12011088 10.1074/jbc.M202883200

[87] Parker MG and Mainwaring WI. Effects of androgens on the complexity of poly(A) RNA from rat prostate. Cell.1977;12(2), pp.401–407. 912750 10.1016/0092-8674(77)90116-7

[88] Watson G, Paigen K. Progressive induction of mRNA synthesis for androgen-responsive genes in mouse kidney. Mol Cell Endocrinol.1990;61, 67–74. 10.1016/0303-7207(90)90171-4 2303161

[89] Toole JJ, Hastie ND, Held WA. An abundant androgen-regulated mRNA in the mouse kidney.Cell.1979; 6 17, 441–8. 455472 10.1016/0092-8674(79)90170-3

[90] Sheflin LG, Zou AP, Spaulding SW.Androgens regulate the binding of endogenous HuR to the AU-rich 3’UTRs of HIF-1 and EGF mRNA. BiochemBiophys Res Commun.2004;322,644–651. 10.1016/j.bbrc.2004.07.173 15325278

[91] Dong X, Sweet J, Challis JRG, Brown T, Lye SJ. Transcriptional activity of androgen receptor is modulated by two RNA splicing factors, PSF and p54nrb. Mol Cell Biol.2007;27,4863–4875. doi: 10.1128/MCB.02144-06 17452459 10.1128/MCB.02144-06 PMC1951499

[92] Wegner M, Stolt CC.From stem cells to neurons and glia: a Soxist's view of neural development.Trends Neurosci.2005; 11;28(11):583–8. doi: 10.1016/j.tins.2005.08.008 16139372 10.1016/j.tins.2005.08.008

