# Supplementary figures and images for: Transcriptional role of androgen receptor in the expression of long non-coding RNA Sox2OT in neurogenesis
Source: PLoS One. 2017 Jul 12;12(7):e0180579. doi: 10.1371/journal.pone.0180579 (PMC5507538; doi:10.1371/journal.pone.0180579)

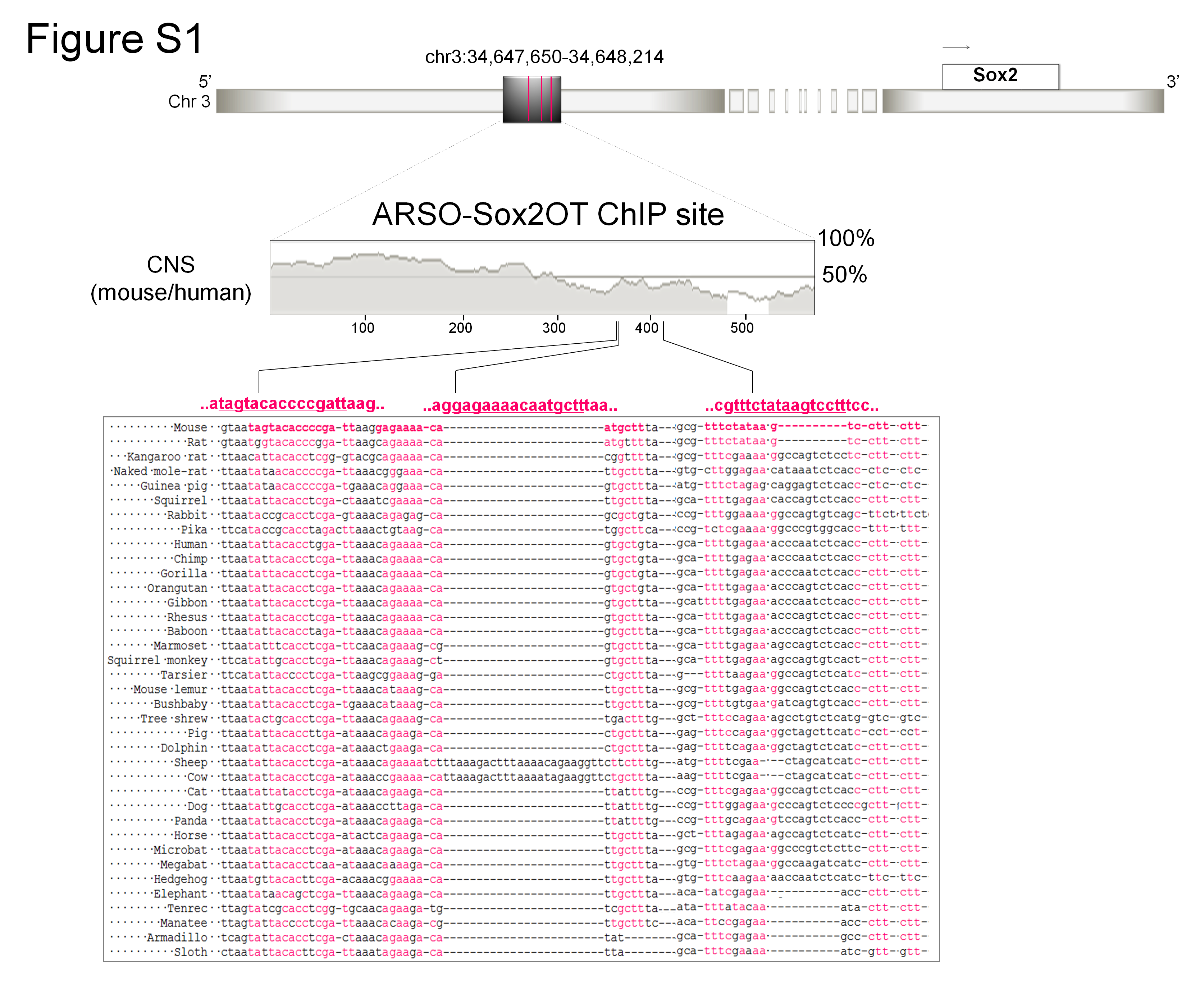

Supplement: S1 Fig — White box, Sox2 gene; gray boxes, region tested by ChIP with ChIP-grade antibodies; vertical red lines, AR consensus sites (ARE). The direction of Sox2 gene transcription is shown with arrow. The phylogenetic conservation (CNS) Mouse-human of ARSO-Sox2OTsequence is shown below the gene diagram (derived from the VISTA browser). High sequence conservation of ARE (UCSC browser) at aligned sites across the species (shown at the left). Identical nucleotides are shown in the color pink. (TIF) [file pone.0180579.s001.tif]

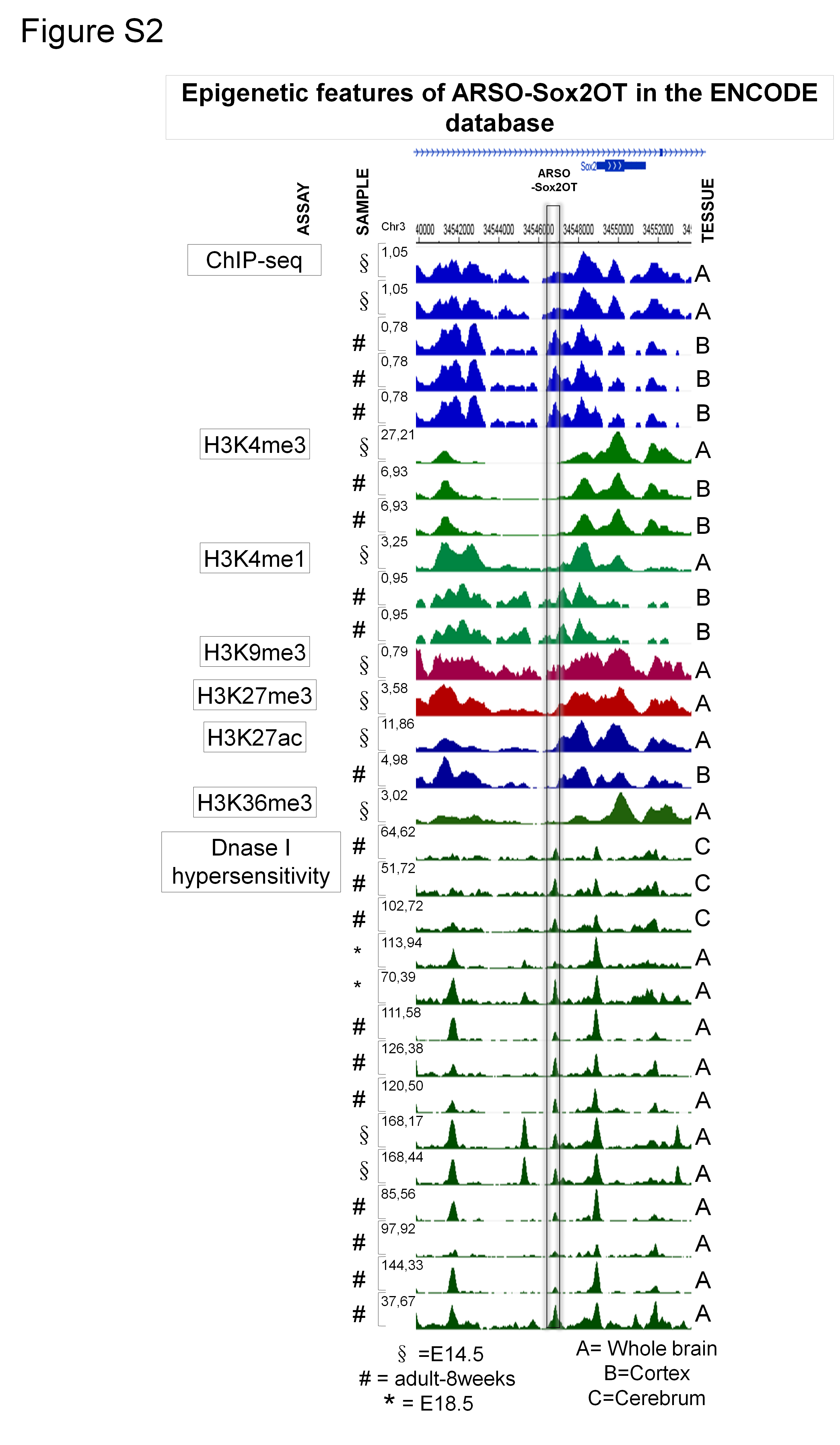

Supplement: S2 Fig — Integration at ARSO-Sox2OT site of ENCODE high-throughput experiments performed in mouse cerebral tissues. Respect to Sox2 locus at top, vertical rectangular shows the ARSO-Sox2OTchromatin region. From top to bottom, row reports the genomic coverage obtained by DNAse-Seq and ChIP-Seq against different histone modifications. Letters on the right column indicate the tissues analyzed, row by row. Sample information isreported at bottom. Top panel shows results obtained by ENCODE Open Chromatin by DNase-Duke University and Open Chromatin by DNase-Washington University. Is evident a peak in the ARSO-Sox2OT sequence, which is more present in embryonic than adult tissues.H3K4me3/1, H3K36me3, and H3K27ac are modifications associated with active chromatin and H3K27me3 and H3K9me3 for silenced heterochromatic region. The image was generated using Wash U Epigenome Browser. (TIF) [file pone.0180579.s002.tif]
